# DNA Methylation Profiling of the Human Major Histocompatibility Complex: A Pilot Study for the Human Epigenome Project

**DOI:** 10.1371/journal.pbio.0020405

**Published:** 2004-11-23

**Authors:** Vardhman K Rakyan, Thomas Hildmann, Karen L Novik, Jörn Lewin, Jörg Tost, Antony V Cox, T. Dan Andrews, Kevin L Howe, Thomas Otto, Alexander Olek, Judith Fischer, Ivo G Gut, Kurt Berlin, Stephan Beck

**Affiliations:** **1**The Wellcome Trust Sanger Institute, HinxtonCambridgeUnited Kingdom; **2**Epigenomics AGBerlinGermany; **3**Centre National de GénotypageEvry CedexFrance

## Abstract

The Human Epigenome Project aims to identify, catalogue, and interpret genome-wide DNA methylation phenomena. Occurring naturally on cytosine bases at cytosine–guanine dinucleotides, DNA methylation is intimately involved in diverse biological processes and the aetiology of many diseases. Differentially methylated cytosines give rise to distinct profiles, thought to be specific for gene activity, tissue type, and disease state. The identification of such methylation variable positions will significantly improve our understanding of genome biology and our ability to diagnose disease. Here, we report the results of the pilot study for the Human Epigenome Project entailing the methylation analysis of the human major histocompatibility complex. This study involved the development of an integrated pipeline for high-throughput methylation analysis using bisulphite DNA sequencing, discovery of methylation variable positions, epigenotyping by matrix-assisted laser desorption/ionisation mass spectrometry, and development of an integrated public database available at http://www.epigenome.org. Our analysis of DNA methylation levels within the major histocompatibility complex, including regulatory exonic and intronic regions associated with 90 genes in multiple tissues and individuals, reveals a bimodal distribution of methylation profiles (i.e., the vast majority of the analysed regions were either hypo- or hypermethylated), tissue specificity, inter-individual variation, and correlation with independent gene expression data.

## Abstract 2

DNA is frequently modified by methylation, which can affect its function. The Human Epigenome Project aims to identify, catalog, and interpret DNA methylation throughout the genome

## Introduction

DNA methylation is indispensable for vertebrate genome function. It is involved in diverse genomic processes such as gene regulation, chromosomal stability, and parental imprinting (Bird 2002), and interest in the function of DNA methylation is further heightened by the various human diseases associated with epigenetic dysfunction, a notable example being cancer (Laird 2003). However, the DNA methylation profile of the human genome is still largely a mystery.

The sequencing of the human genome (IHGSC 2001) and creation of a whole-genome map of single nucleotide polymorphisms (SNPs) (Sachidanandam et al. 2001) laid the foundation for the Human Epigenome Project (HEP). For the HEP, we aim to analyse DNA methylation in the regulatory regions of all known genes in most major cell types and their diseased variants, along with producing high-density snapshots of non-genic regions spread evenly across the human genome. Although genome-wide DNA methylation analyses have been performed previously (Costello et al. 2000; Strichman-Almashanu et al. 2002), the HEP is the first systematic whole-genome study of DNA methylation at the sequence level.

As a prelude to the HEP, here we report the results of the HEP pilot study: DNA methylation profiling of the human major histocompatibility complex (MHC). The MHC, located on Chromosome 6 (6p21.3), is one of the most gene-dense regions in the human genome, containing genes with a high diversity of function, many of which are involved in the innate and adaptive immune systems. We chose to analyse the MHC for the pilot HEP study for three main reasons. (i) The MHC is associated with more diseases than any other region of the human genome, and therefore the generated data will be of interest to researchers with diverse biomedical interests. (ii) It is also the most polymorphic region in the genome, and therefore the data will allow study of the potential effects of the loss or gain of cytosine–guanine dinucleotide (CpG) methylation sites (due to SNPs) on gene expression and possibly other phenotypes. (iii) At the time when the HEP pilot study was initiated in 1999 (Beck et al. 1999), the MHC was one of the few regions within the human genome for which finished sequence and annotation were readily available (MHC Sequencing Consortium 1999).

Using an integrated pipeline involving high-throughput bisulphite DNA sequencing, we have determined the DNA methylation levels within the vicinity of the promoter and other relevant regions, such as CpG islands and first exons and introns of 90 genes within the 3.8-Mb MHC region in multiple tissues and individuals. Our analysis reveals a bimodal distribution of methylation levels, tissue specificity, and inter-individual variation. We have also developed matrix-assisted laser desorption/ionisation mass spectrometry (MALDI-MS) assays for high-throughput epigenotyping of the analysed regions. Finally, we have established a publicly available database for the HEP data (http://www.epigenome.org), which integrates, for the first time, epigenetic information with the existing genome annotation.

## Results/Discussion

For the DNA methylation profiling of the human MHC, the following regions of interest (ROIs) were chosen: (i) a potential regulatory region for each gene and (ii) the most CpG-dense region of each gene. It is well established that epigenetic modifications at regulatory regions, in particular promoters, correlate with the transcriptional state of the cognate gene (reviewed in Bird 2002). Because the precise locations of promoters within the human MHC were unknown at the time this study was initiated, we surmised that analysing a region from 2 kb upstream to 500 bp downstream of the annotated start codon would, in many cases, include the promoter region. Such regions were designated as “upstream” ROIs. ROIs representing the most CpG-dense region within each gene were defined for the region from 500 bp downstream of the annotated start codon to the end of the gene and did not exceed a total length of 2.5 kb. These ROIs were named “intragenic”. For longer genes, more than one intragenic ROI was chosen. Within each ROI, we used the amplicon with the highest CpG density that could be successfully amplified. Other amplicons, if used, were chosen based on the ranking of their CpG density. Wherever possible, CpG islands associated with genes were included. (CpG islands have been defined by Bird [1986] as a contiguous window of DNA of at least 200 bp in which the G + C content is at least 50% and the ratio of observed over expected CpG frequency is greater than 0.6. We used a slightly stricter definition: regions of at least 400 bp in which the G + C content is at least 50% and the ratio of observed over expected CpG frequency is greater than 0.6.) All known repeat sequences were avoided during amplicon design. Methylation was analysed in seven human tissues—adipose, brain, breast, liver, lung, muscle, and prostate—with multiple samples from different individuals for all tissues (except adipose) (see Table S1).

**Figure 1:**
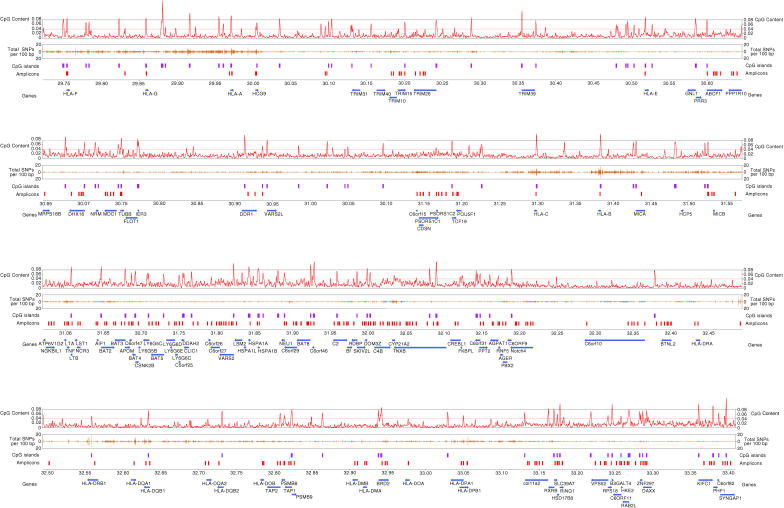
Map of the Human MHC Showing Coverage and Locations of the Bisulphite PCR Amplicons for Which Methylation Data Have Been Generated Tracks from top to bottom are as follows. (1) CpG content—the proportion of CpGs in 8-kb windows. The expected proportion of CpG dinucletides is 0.04 based on the background base composition of Chromosome 6 (Mungall et al. 2003). (2) Random SNP density in 1,000-bp windows. (3) Location of predicted CpG islands. (4) Bisulphite PCR amplicons. (5) Location of annotated gene structures. Right and left arrows indicate gene structures on the sense and antisense strand, respectively. Official gene symbols are used where available.

Figure 1 shows the locations and the coverage provided by the bisulphite PCR amplicons across the 3.8-Mb human MHC in the context of annotated genes, CpG content, CpG islands, and SNPs extracted from the SNP database (http://www.ncbi.nlm.nih.gov/SNP). A total of 253 unique amplicons were successfully analysed (Table 1). On average, the amplicons were 438 bp in length (which is close to the optimum amplicon length for the bisulphite PCR), were relatively GC-rich (average G + C > 50%), and had a high density of CpGs (approximately 1 CpG/31 bp). Ninety genes (i.e., more than 70% of all expressed genes within the MHC) were represented by at least one amplicon. Of the analysed CpG sites, 80% displayed methylation levels that varied (i.e., by more than 20%) either between individuals and/or tissues, suggesting that the potential information content of the selected amplicons was relatively high.

**Table 1:**
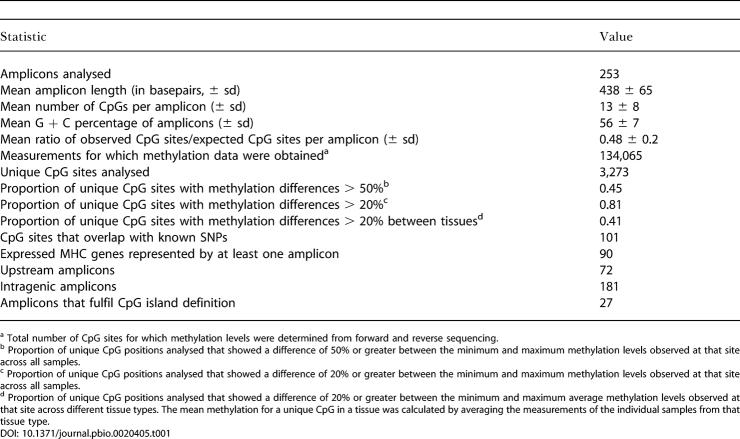
Amplicon Statistics

^a^ Total number of CpG sites for which methylation levels were determined from forward and reverse sequencing

^b^ Proportion of unique CpG positions analysed that showed a difference of 50% or greater between the minimum and maximum methylation levels observed at that site across all samples

^c^ Proportion of unique CpG positions analysed that showed a difference of 20% or greater between the minimum and maximum methylation levels observed at that site across all samples

^d^ Proportion of unique CpG positions analysed that showed a difference of 20% or greater between the minimum and maximum average methylation levels observed at that site across different tissue types. The mean methylation for a unique CpG in a tissue was calculated by averaging the measurements of the individual samples from that tissue type

### Quantification of DNA Methylation by Direct Sequencing of Bisulphite PCR Products

**Figure 2:**
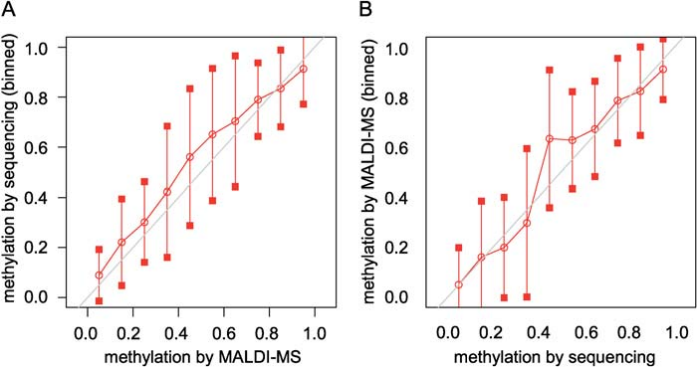
Comparison of Methylation Measurements Obtained Using MALDI-MS with Those from ESME Analysis of Directly Sequenced Bisulphite PCR Products (A) Comparison of methylation measurements obtained by MALDI-MS (x-axis) with ESME-processed data from sequencing (y-axis). Methylation rates at CpGs from forward and reverse sequencing were binned into ten intervals from zero to one using corresponding MALDI-MS measurements at the same CpGs and in the same tissue samples. (B) Comparison of methylation measurements obtained from ESME-processed data (x-axis) with measurements from MALDI-MS (y-axis). Methylation rates from MALDI are binned as in (A), using the corresponding methylation values from sequencing. Red lines show the means of the binned rates; bars show the standard deviations. The overall correlation of the data is 0.887. Data points that are not around a methylation rate of zero or one are covered by few measurements because of the bimodal distribution of methylation measurements.

We analysed DNA methylation using bisulphite sequencing (Olek et al. 1996). In the presence of sodium bisulphite, unmethylated cytosines are converted to uracil, whereas methylated cytosines are unreactive under the same conditions. After bisulphite treatment the DNA is subjected to PCR and sequencing. Methylated cytosines are detected as cytosines in the sequencing reaction, whereas all unmethylated cytosines appear as thymidines. Traditionally, bisulphite PCR analysis involves sequencing multiple sub-clones of the bisulphite PCR product. This approach is time-consuming, and there have also been reports of bias (Grunau et al. 2001) and hetero-duplex amplification (Sandovici et al. 2003) during sub-cloning of bisulphite PCR products. We sequenced the bisulphite PCR products directly with the same primers used in the PCR and developed software, called ESME (Lewin et al. 2004), to determine the DNA methylation levels from the sequence trace files. Briefly, ESME performs quality control, normalises signals, corrects for incomplete bisulphite conversion, and maps positions in the trace file to CpGs in the reference sequence. The program calculates methylation levels by comparing the C to T peaks at CpG sites, with the ability to discriminate levels of methylation that differ by as little as 20%. Methylation estimation by ESME at any given CpG site is the average from all the copies generated during PCR and is, therefore, compared to sub-cloning, a more accurate representation of the methylation level. Furthermore, we reanalysed the methylation levels of 77 amplicons by MALDI-MS, which allows for discrimination of methylation levels that differ by as little as 5% (Tost et al. 2003). Figure 2 shows the comparison of the two methods, demonstrating a concordance rate of 88% between ESME and MALDI-MS.

### The HEP Database

**Figure 3:**
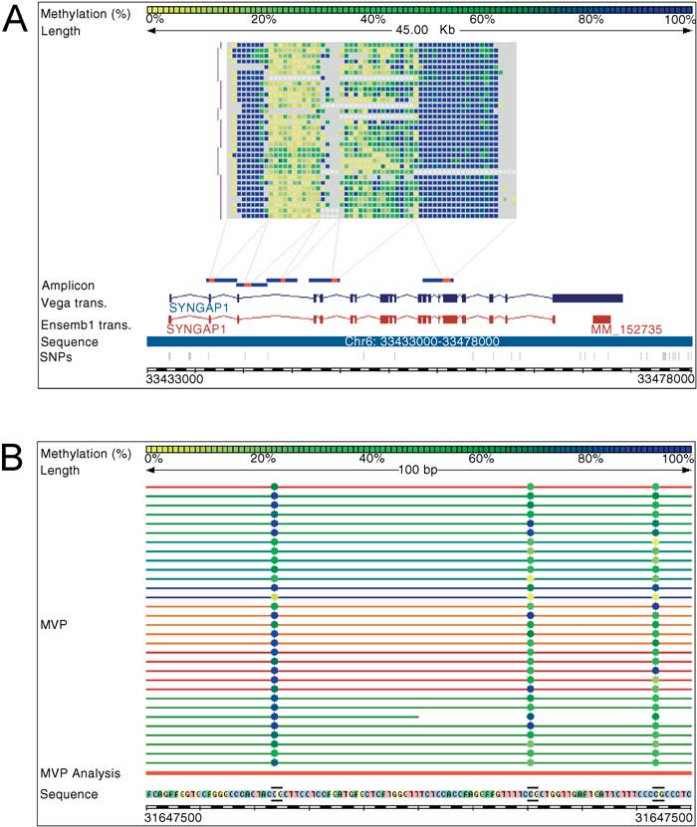
The HEP Database (A) We have created a Web-based, ENSEMBL-like genome browser for displaying HEP data that is publicly available at http://www.epigenome.org. The methylation levels calculated by the ESME software are displayed in the form of a matrix. Each matrix contains the data obtained from all the samples of one amplicon. Each colour-coded square (yellow represents 0% methylation, blue represents 100% methylation, and green represents intermediate levels) within the matrix represents one CpG site. Clicking on a square reveals the tissue source of the sample and the level of methylation observed at that particular CpG site. Grey squares indicate CpG sites for which methylation levels could not be determined. Each row of squares represents all the CpG sites for one sample of a particular amplicon, and the samples are grouped by tissue type. The red bar indicates the genomic region analysed. Also shown are chromosome coordinates, CpG islands, SNPs, and ENSEMBL and high-quality, manually curated VEGA transcript information. The HEP database links to the Ensembl genome browser, providing additional information about the region of interest. The example shows amplicons within the *SynGAP 1* gene that correspond to regions that were determined to be hypomethylated (second amplicon from the left), hypermethylated (first and fifth amplicons), and heterogeneously methylated (fourth amplicon). Insufficient data were obtained for the third amplicon. (B) By using the zoom function, the user can view the complete DNA sequence for the analysed amplicon.

To make the data generated in this study a publicly available resource, we have designed a Web-based, ENSEMBL-like genome browser (http://www.epigenome.org) that allows easy access to the data from the pilot HEP study (Figure 3A and 3B). Methylation levels calculated by ESME are displayed in a colour-coded matrix. Rows represent the averages of forward and reverse sequences for various tissues while columns represent individual CpG sites. Each matrix square therefore represents the average methylation level at a given CpG site for a given tissue. Multiple data rows are available for all tissues (except adipose). Clicking on a square in the genome browser reveals the level of methylation observed at that particular CpG site (the average of the forward and reverse sequence) and information about the tissue source. Additional annotation includes chromosome coordinates, CpG islands, SNPs, ENSEMBL and high-quality, manually curated Vertebrate Genome Annotation database transcripts, the ROIs, and amplicon and primer sequences. The browser provides a zoom function to view the genomic sequence (Figure 3B), and a link to ENSEMBL facilitates access to additional information and the ENSEMBL search engines. The data from the full-scale HEP will be made available via the same browser, providing a novel public resource for the research community.

### Methylation Profile Characteristics of the MHC

**Figure 4:**
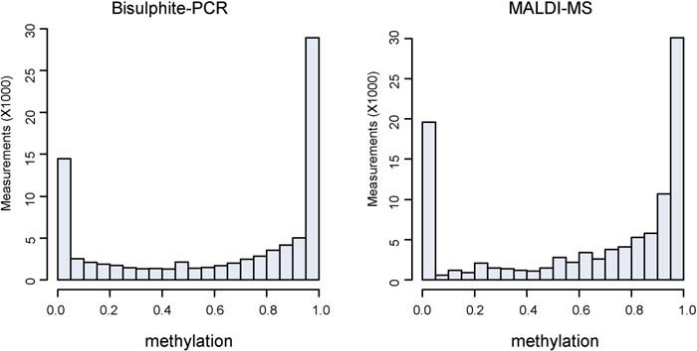
Bimodal Distribution of DNA Methylation within the Human MHC (A) Determined by direct sequencing/ESME analysis (based on 86,374 single CpGs in different tissue samples building the median for measurement repetitions). (B) Determined by MALDI-MS (based on 1,019 MALDI measurements).

The methylation profile of the human MHC region appears to be strongly bimodal, with over 90% of the amplicons being either relatively hypomethylated (i.e., median methylation of amplicon 30% or less) or relatively hypermethylated (i.e., median methylation of amplicon 70% or greater) (Figures 3A and 4). Re-analysis of a subset of the data by MALDI-MS confirmed the bimodality of the methylation profile (Figure 4). Extensive bimodality of genomic methylation profiles has been observed by several authors (reviewed in Bird 2002). Furthermore, the experiments of Lorincz et al. (2002) suggest that the extremes of methylation profiles may in fact be the most stable states within the genome. Lorincz et al. showed that a high density of methylation at a proviral construct is stably propagated in vivo, whereas a low density of proviral methylation is inherently unstable, with daughter cells harbouring proviral cassettes that are demethylated or de novo methylated. It must be noted that even though the amplicons displayed hypo- or hypermethylated profiles, small variations in the levels of methylation at individual CpG sites within an amplicon were also frequently observed. Although there may be technical reasons for this heterogeneity, numerous studies (using a variety of techniques) have shown that the methylation profile of a given region in vivo is rarely homogenous (Costello et al. 2000; Kondo et al. 2000; Grunau et al. 2001; Cui et al. 2003). The functional outcome of these small variations, particularly when they exist between tissues or individuals, remains to be elucidated.

Comparison of the methylation values for upstream amplicons (median methylation of 10%) versus intragenic amplicons (median methylation of 86%) revealed that upstream amplicons were more likely to be hypomethylated (*p* < 0.0001). Interestingly, within the upstream category we found that CpG sites located within the 5′ UTR were less likely to be methylated (median methylation of 7%) than the CpG sites located within 2 kb of the first start codon but not within the 5′ UTR (median methylation of 14%) (*p* < 0.0001). Within the intragenic category, we found that CpG sites located within introns (median methylation of 84%) were less likely to be methylated than CpG sites located within exons (median methylation of 89%) (*p* < 0.0001). Whether these significant but small differences reflect any bias for the presence of regulatory elements close to the transcriptional start site or within introns, or some other functional consequence, is currently hard to assess.

### Analysis of Heterogeneously Methylated Regions

Fourteen amplicons displayed significant heterogeneous methylation profiles (i.e., median methylation between 30% and 70%) (see Figure 3A). These might represent differentially methylated regions at which parental alleles display reciprocal methylation profiles that are determined by the parent-of-origin of the allele, or regions that were heterogeneously methylated on both alleles. Our sequencing method could not discriminate between these two possibilities, and none of these regions corresponded to known imprinted sites within the human genome. We therefore sub-cloned the PCR products and sequenced individual sub-clones of ten different heterogeneously methylated amplicons and used polymorphisms to discriminate between the parental alleles. The overall methylation profiles determined by sequencing individual sub-clones were consistent with those obtained by direct sequencing of the bisulphite PCR products. None of the six amplicons for which polymorphisms were found showed allele-specific methylation (data not shown). This is consistent with the fact that so far there have been no reports of imprinted regions within the human MHC. Since these amplicons were heterogeneously methylated to a similar extent in samples from various tissues and individuals, they might represent regions where maintenance of a specific epigenetic state is not essential. It is also possible that these regions are located at the boundaries of hypermethylated regions, and, consequently, the methylation levels are “trailing off”. However, both these possibilities contradict models that suggest that the genome prefers to maintain methylation profiles in bimodal states.

Interestingly, a few regions were heterogeneously methylated in some tissues only, suggesting that the tissue sampled was a mosaic of several sub-types among which the methylation profile at certain genes varied, or that the region displays tissue-specific parental imprinting similar to the *insulin-like growth factor 2 (IGF2)* gene, which is imprinted in all tissues except brain (Pham et al. 1997).

Direct sequencing of the heterogeneously methylated amplicons was unsuccessful in a small proportion of cases. Possible reasons include incomplete bisulphite conversion and genetic polymorphisms within the primer binding site. We also noticed a mobility shift of the sequence in a few cases. This occurs because a population of bisulphite PCR products generated from a heterogeneously methylated region contains a mixture of molecules, some with cytosines at certain CpG sites (i.e., initially methylated) and others with thymidines at those CpG sites (i.e., initially unmethylated). When these PCR products are sequenced directly, the cumulative effect of the molecular weight difference between cytosines and thymidines is that some molecules migrate faster than others during capillary electrophoresis. The sequence trace therefore contains two traces that do not perfectly overlap, resulting in erroneous estimation of methylation levels. Such sequences were excluded from further analyses.

### Analysis of CpG Islands

CpG islands are GC-rich regions that contain a high density of CpGs and are positioned at the 5′ ends of many human genes (reviewed in Bird 2002). Although most CpG islands remain hypomethylated throughout development in all tissues (Antequera and Bird 1993), regardless of expression state, a small proportion become hypermethylated during development (reviewed in Bird 2002), and this correlates with transcriptional silencing of the associated gene. In our study, 27 amplicons overlapped CpG islands, and 22 of these (i.e., 80%) were hypomethylated in all tissues examined. Interestingly, this proportion of hypomethylated CpG islands is similar to that reported by Yamada et al. (2004), who analysed the methylation status of CpG islands on human Chromosome 21q and found that 103 out of 149 CpG islands (i.e., 70%) were hypomethylated.

In our study, CpG island amplicons situated in the upstream ROIs were always hypomethylated, whereas hypermethylated CpG island amplicons were found only in the intragenic regions. Among the intragenic CpG island amplicons, those situated at the 5′ end of the gene (i.e., overlapping exon 1, intron 1, or exon 2) were always hypomethylated. A tissue-specific methylation profile was observed for the CpG island situated within exon 3 of the *tenascin-XB* (*TNXB*) gene, which was hypomethylated in muscle samples only. This hypomethylation correlates with the temporally regulated and tissue-specific expression of *TNXB,* which is abundantly expressed in connective tissues. It has been suggested that *TNXB* has a role in limb, muscle, and heart development (Burch et al. 1995), and, therefore, epigenetic modifications at the *TNXB* CpG island may have an important regulatory role (tissue specificity of methylation profiles is discussed in more detail below). Interestingly, the CpG island amplicon located within exon 3 of the *HLA-G* gene spanned a methylation boundary, being hypomethylated at the 5′ end with a sharp transition to a hypermethylated profile at the 3′ end. Overall, the results are consistent with the prevailing model of CpG islands being regions of the genome that are hypomethylated, especially when they occur upstream or within the 5′ end of the gene.

### Tissue Specificity of the Methylation Profiles

DNA methylation profiles are complex and dynamic, and can vary with developmental stage, tissue type, age, the alleles' parent-of-origin, and also phenotype or disease state (reviewed in Bird 2002). In particular, the role of DNA methylation in setting up and maintaining tissue-specific expression patterns has received a lot of attention. However, the extent of tissue specificity of DNA methylation profiles is relatively unknown. The HEP pilot study involved the analysis of 32 samples (from different individuals) comprising seven tissues: adipose, brain, breast, liver, lung, muscle, and prostate.

**Figure 5:**
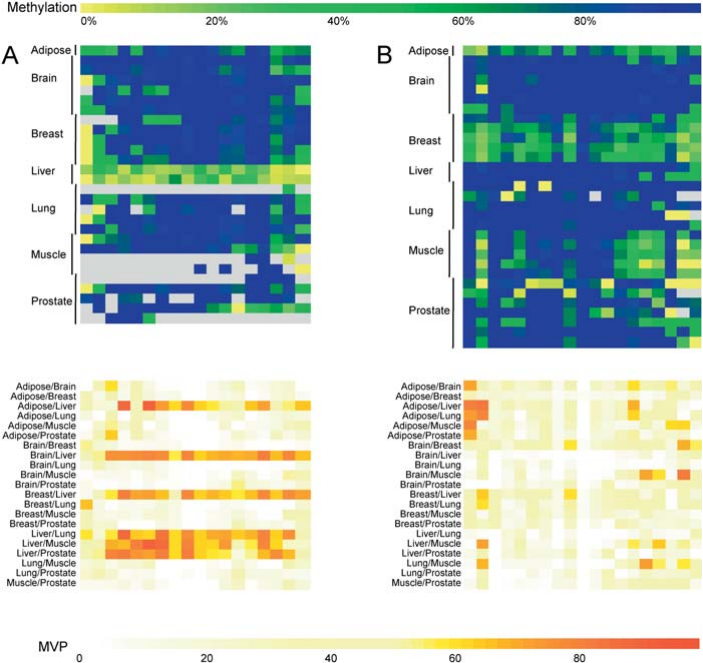
Example of METHANE Output Showing Regions That Display Tissue-Specific Methylation Profiles The top colour-scale bar refers to the degree of methylation (percent). The bottom colour-scale bar refers to the absolute difference in the methylation level observed between tissues at a given CpG site, and is therefore a measure of the confidence level for a CpG site to be defined as a MVP. (A) The upper matrix represents an amplicon that contains 18 CpG sites within a 386-bp region overlapping exon 3, intron 3, and exon 4 of the *complement factor B* gene. It is hypomethylated in liver (median methylation is 17%) and hypermethylated in all other tissues examined (median methylation is 100%). The lower matrix shows pairwise comparisons of the methylation values for each CpG site between tissues. (B) The upper matrix represents an amplicon that contains 19 CpG sites within a 550-bp region overlapping exon 3 and intron 3 of the *DAXX* gene. It is relatively hypomethylated in breast (median methylation is 64%) compared with the other tissues examined (median methylation is 100%). The lower matrix shows pairwise comparisons of the methylation values for each CpG site between tissues.

Upon comparison of the amplicon profiles, we found that 10% of all amplicons displayed differential methylation between the tissue types (examples are shown in Figure 5). Of these amplicons, 31% were located in the upstream regions, a proportion that is in the same range as the total number of upstream amplicons relative to intragenic amplicons analysed in this study (see Table 1). We scanned the literature and publicly available gene expression databases to determine whether the cognate genes displayed tissue-specific expression. An example is the complement protein *C2* mRNA, which normally has a long 5′ upstream region; in the liver, an additional transcript with a much shorter 5′ upstream region is expressed (Horiuchi et al. 1990). In our study we found that a region that overlaps intron 2 and exon 2 of the *C2* gene was hypomethylated in liver samples only (however, this region is downstream of the transcriptional start sites of both forms of *C2* mRNA). Another example is *DOM3Z,* which is ubiquitously expressed but occurs only at very low levels in the lung (Yang et al.1998), and this correlates with a region overlapping exons 4 and 5 of *DOM3Z* that is hypermethylated in lung (and brain) but hypomethylated in the other tissues examined. It has also been demonstrated that the murine *complement factor B* utilises differential tissue-specific start sites (Garnier et al. 1995), and in our analysis the human homologue is hypomethylated at a region overlapping exons 3 and 4 only in liver. However, the majority of the genes that were associated with tissue-specific methylation profiles in our study did not show corresponding tissue-specific expression profiles in a previously reported whole human genome expression microarray analysis (Su et al. 2002). Some of these genes are known to be associated with various mRNA isoforms, but detection of such alternative transcripts is quite difficult with conventional microarray analysis and usually requires more detailed analysis. It is also possible that the tissue-specific methylation profiles we observed in adult tissue may hint at tissue-specific expression profiles that existed during early development, or they may be associated with as yet unknown transcripts, e.g., non-coding RNAs. Alternatively, there may be only a modest proportion of genes in which tissue specificity of gene expression is affected by methylation.

### Inter-Individual Variation of Methylation Profiles

There is increasing evidence that an individual's epigenetic profile can influence phenotype and susceptibility to various diseases such as cancer, an example of such evidence being a recent report linking the loss of imprinting at the *IGF2* locus with an increased risk of developing colorectal cancer (Cui et al. 2003). In our study, nearly all loci displayed some degree of heterogeneity, which probably has no bearing on the differences in genome function among individuals. However, considerable differences in methylation profiles between individual samples within a tissue were observed for a number of amplicons. We calculated a median methylation value for each individual sample and then compared these values within each tissue type for each amplicon. A total of 118 amplicons displayed a difference of greater than 50% between the lowest and highest median methylation values in at least one tissue. Of these amplicons, 76% were intragenic, which is a similar proportion to the overall number of intragenic amplicons (71%; 181 out of 253 amplicons) analysed in the study. This proportion is also similar to the overall proportion of amplicons that showed tissue-specific methylation profiles and were classified as intragenic (69%). Although inter-individual variation for a given amplicon was not observed in every tissue, there was no apparent tissue-specific enrichment for inter-individual variability of methylation profiles.

**Figure 6:**
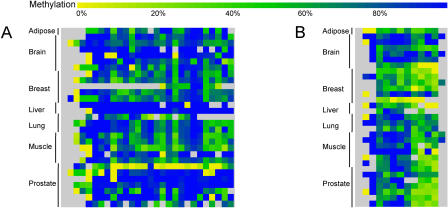
Example of METHANE Output Showing Regions That Display Inter-Individual Variation of Methylation Profiles (A) Example of a region that displays significant inter-individual variation, especially in prostate. The matrix represents an amplicon that contains 27 CpG sites within a 527-bp region overlapping the last exon of the *CYP21A2* gene. (B) Another example of a region that displays significant inter-individual variation. The matrix represents an amplicon that contains 13 CpG sites within a 453-bp region overlapping the 5′ UTR and exon 1 of the *tumour necrosis factor* gene.

Examples of amplicons that displayed significant inter-individual variation in methylation profiles include a region overlapping the last exon in *CYP21A2* that showed considerable inter-individual variation in prostate (Figure 6A), and a 5′ upstream region of *tumour necrosis factor* (LocusID 7124) that varied significantly between individuals in liver (Figure 6B). Although the differences could be attributable to the technical variability inherent in our approach or the fact that we did not control for age or sex of the tissue donors, it is also possible that certain genotypes are associated with unique epigenotypes. In a recent study, Van Laere et al. (2003) mapped a porcine quantitative trait locus that affects muscle growth, fat deposition, and heart size to an evolutionarily conserved CpG island within the imprinted *Igf2* gene. Pigs inheriting the mutation from their sire had a 3-fold increase in *Igf2* expression in postnatal muscle (i.e., the quantitative trait locus is paternally expressed). Furthermore, the mutation abrogated in vitro interaction with a nuclear factor, and this effect was phenocopied following in vitro DNA methylation of the region. Evidence for an interaction between genotype and epigenotype at the *IGF2* gene in humans has also recently been reported (Murrell et al. 2004). Of the 3,273 unique CpG sites we analysed, 101 overlapped with known SNPs (relatively evenly distributed over all amplicons), all representing sites at which the CpG was lost (see Figure 1; Table 1). The SNPs were extracted from dbSNP (http://www.ncbi.nlm.nih.gov/SNP) and are annotated in the HEP database. One could postulate that the gain or loss of one or more critical CpG sites may affect the overall methylation profile of a locus and, consequently, promoter activity. Alternatively, non-CpG SNPs located within an epigenetically sensitive regulatory element could also influence the epigenetic makeup of that region. Therefore, mutations in regulatory sequences could influence epigenetic profiles, resulting in altered phenotypes.

### Analysis of Methylation Variable Positions by MALDI-MS

**Figure 7:**
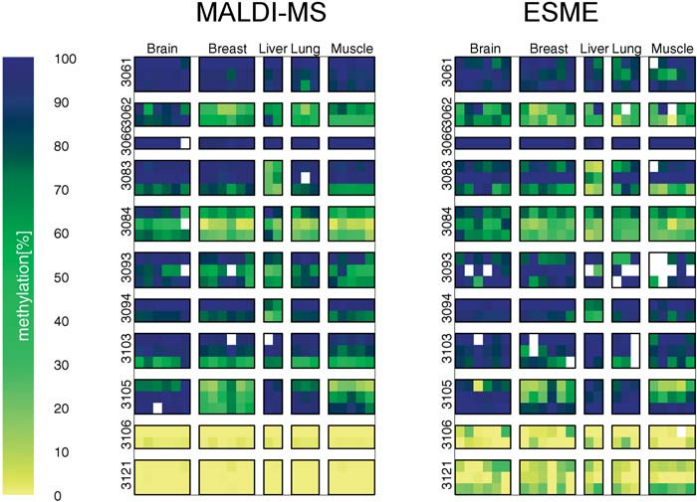
Comparison of Methylation Values Measured in Five Tissues and Eleven Amplicons Using MALDI-MS and ESME Analysis of Directly Sequenced PCR Products Each column is a tissue sample, each row a CpG site. Data are ordered in blocks by tissue type and amplicons. Positions of measurements for MALDI-MS (A) correspond to those for ESME analysis (B). The methylation values are colour coded from 0% methylation (yellow) to 100% methylation (blue), with intermediate methylation levels represented by shades of green. White indicates missing measurement values.

A major aim of the HEP is to identify genomic regions at which DNA methylation profiles display statistically significant variation due to biological or environmental influences. Therefore, based on the tissue-specific and inter-individual variation in methylation profiles discussed above, we were interested in establishing high-throughput assays for epigenotyping. This involved the identification (manually or using the METHylation ANalysis Engine [METHANE]) of methylation variable positions (MVPs), which we define as CpG sites that have statistical power to discriminate between different biological samples or states. In other words, by assaying the methylation state of just a few select CpG sites within a given region, information can be inferred about the tissue source or disease state. Such a high-throughout MVP epigenotyping method was recently developed based on the GOOD assay (Tost et al. 2003). This recently developed epigenotyping assay allows for accurate discrimination of methylation levels that differ by 5% or more. Furthermore, MALDI-MS is a relatively inexpensive method that offers a high degree of automation and integration and that has no requirement for sample purification. Assays for 231 MVPs in 77 amplicons, including all those that displayed differential methylation profiles between different tissue types or inter-individual variability, were designed and analysed in a triplex format (i.e., methylation levels at three independent CpG sites are analysed in one assay). A subset of 11 MALDI-MS assays is shown in Figure 7.

### Comparison of Methylation Profiles with Independent Gene Expression Data

The primary function of epigenetic modifications is to modulate gene expression: a specific combination of epigenetic modifications at regulatory elements, notably promoters and enhancers, influences the transcriptional state of a gene (reviewed in Bird 2002). In many cancers, aberrant epigenetic modifications occur within CpG islands that overlap promoters (some of which are candidate tumour suppressors), which is thought to result in aberrant transcription of the cognate gene, thus contributing to tumour progression.

**Figure 8:**
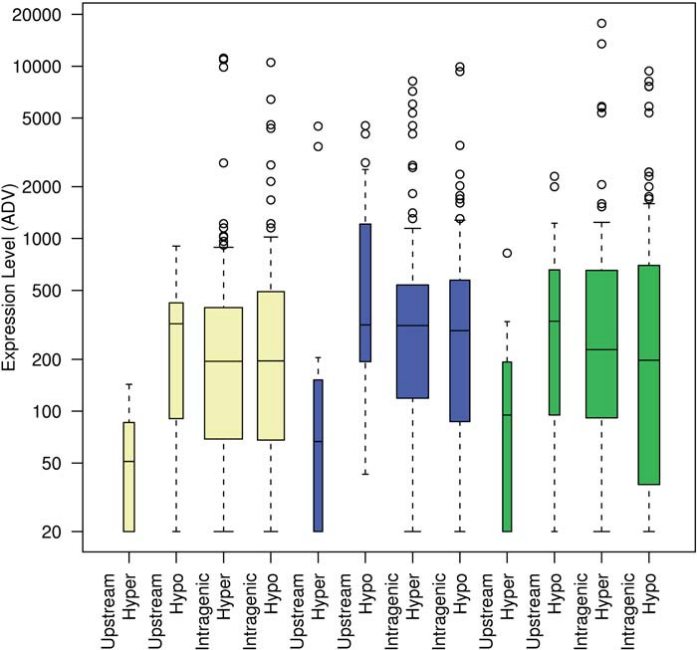
Comparison of DNA Methylation with Gene Expression Amplicons generated from prostate (yellow), lung (blue), and liver (green) samples were divided into two categories: “upstream” and “intragenic”. The median methylation values for the amplicons were calculated as described in the text, and these were then classified as hypomethylated (median methylation less than 50%) or hypermethylated (median methylation greater than 50%), and plotted against the cDNA microarray expression data available at http://expression.gnf.org (Su et al. 2002). The expression values are expressed as average difference values (ADVs) for each gene. The average difference value is computed using Affymetrix software and is proportional to mRNA content in the sample, with a value of 200 being a conservative cut-off below which a gene can be classified as being not expressed. The average difference values are the mean of 2 or 3 independent experiments. For prostate and liver, the expression levels associated with the hypermethylated upstream amplicons were significantly lower than the expression levels associated with the hypomethylated upstream amplicons (*p* < 0.0001 for prostate and *p* < 0.01 for liver). For lung, there was no significant difference between the expression levels associated with the hypermethylated upstream amplicons and those of the hypomethylated upstream amplicons (*p* > 0.3). There was no correlation between expression and methylation for the intragenic amplicons for any of the three tissues (*p* > 0.3). The width of the bars is indicative of the number of amplicons in each category: prostate upstream, hypermethylated (*n* = 9); prostate upstream, hypomethylated (*n* = 15); prostate intragenic, hypermethylated (*n* = 109); prostate intragenic, hypomethylated (*n* = 53); liver upstream, hypermethylated (*n* = 9); liver upstream, hypomethylated (*n* = 14); liver intragenic, hypermethylated (*n* = 115); liver intragenic, hypomethylated (*n* = 45); lung upstream, hypermethylated (*n* = 9); lung upstream, hypomethylated (*n* = 13); lung intragenic, hypermethylated (*n* = 112); and lung intragenic, hypomethylated (*n* = 57).

We compared the amplicon methylation profiles with the human genome expression patterns available from the Genomics Institute of the Novartis Research Foundation Gene Expression Atlas database (http://expression.gnf.org). This publicly available database contains whole-genome mRNA expression data obtained by Su et al. (2002) using human U95A Affymetrix microarray chips. We calculated a median methylation value for each amplicon (see Materials and Methods). As mentioned above, the methylation profiles displayed a bimodal distribution, with more than 90% of the amplicons being either hypomethylated (median methylation of 30% or less) or hypermethylated (median methylation of 70% or greater). Therefore, to perform the analyses we divided the amplicons into two categories: hypomethylated (methylation less than 50%) and hypermethylated (methylation greater than 50%) (see Materials and Methods). We then compared the range of expression values associated with hypomethylated amplicons with those of hypermethylated amplicons. Most genes on the U95 microarray are represented by multiple probes, and, in a few cases, contradictory expression values were obtained for the same gene, in which case the gene was excluded from our analyses. Analyses were performed for liver, lung, and prostate samples only (Figure 8), since appropriate Gene Expression Atlas data were unavailable for the other tissues. For prostate and liver, a significant difference was found between expression levels associated with hypomethylated versus hypermethylated upstream amplicons: hypomethylated upstream amplicons correlated with a wide range of expression levels whereas hypermethylated upstream amplicons correlated with a lack of expression (*p* < 0.0001 for prostate and *p* < 0.01 for liver). The intragenic amplicons did not show any correlation between methylation and expression levels (*p* > 0.3 for both prostate and liver). A list of all upstream amplicons included in the analysis is given in Table S2.

For the lung samples there was no significant correlation between expression and methylation state for amplicons within the upstream or intragenic categories (*p* > 0.3 for both categories). Although the lung data show the same trend as the prostate and liver data, the lung hypomethylated data contained a number of outlier data points representing very high expression values (as shown in Figure 8 by the unfilled circles). The overall trend of the data suggests that these data may be artefactual, but there is nothing that indicates these data points are not real. These data points were enough to influence the analysis such that we could not find a significant difference in the expression of between hypo- and hypermethylated lung genes. If the data points are real, the lack of correlation for the lung samples may be due to inconsistencies within the expression or methylation datasets for lung. Alternatively, there may be additional regulatory elements that influence the expression state of the analysed genes in the lung.

Overall, the findings are consistent with a model in which the DNA methylation profile of the upstream region of the gene is an informative indicator of the expression of the cognate gene, specifically, in which hypermethylation within the upstream region is associated with transcriptional silencing. Furthermore, the data also suggest that epigenetic modifications within the upstream regions influence the transcriptional state of a significant number of the genes within the MHC. This is supported by the study of Jackson-Grusby et al. (2001) in which they employed homogeneous cultures of primary mouse embryonic fibroblasts and used the Cre-loxP system to conditionally inactivate Dnmt1, an enzyme that methylates DNA. They found that in the absence of Dnmt1, several mouse MHC class I genes showed altered expression profiles.

### Concluding Remarks

One of the principal challenges in the post-genomic era is to provide a holistic view of genome function, a challenge which is currently being addressed by several large-scale studies of the transcriptome, proteome, metabolic networks, and haplotype maps. The HEP is therefore timely, since DNA methylation is an indispensable part of the genome's regulatory mechanisms. Here we have described the pilot study for the HEP—DNA methylation profiling of the MHC region—which is the first systematic large-scale study of methylation profiles at the sequence level within a multi-megabase region of the human genome. For this project, we developed an integrated pipeline for high-throughput methylation analysis using bisulphite DNA sequencing, MVP discovery, and epigenotyping by MALDI-MS, and created an integrated database (http://www.epigenome.org) for public access to the data generated by the study. The results from the pilot study demonstrate that a significant proportion of the analysed loci within the MHC show tissue-specific methylation profiles, and inter-individual methylation differences are common. Furthermore, the tissue-specific differences in DNA methylation suggest that epigenetic mechanisms are involved in the use of alternative transcriptional start sites. We have also shown that the generated methylation data allow the identification of MVPs that can be typed with high quantitative resolution and sensitivity using MALDI-MS, providing a tool for large population-based studies and for diagnosing diseases in the future.

The study reported here lays the foundation for the HEP, which aims to analyse the methylation state of the regulatory regions of all annotated genes in most major cell types and their diseased variants. In the first phase, which is well underway, we are analysing the DNA methylation profiles of over 5,000 amplicons (representing a 20-fold scale-up relative to the pilot HEP study reported here) associated with nearly all the annotated genes (approximately 3,000) on human Chromosomes 6, 13, 20, and 22. The excellent genomic annotation available for these four chromosomes, e.g., high-quality transcript information and location of SNPs, will enable us to perform comprehensive analyses linking the epigenetic information gained from the HEP with the underlying genetic information. Samples from over 40 different individuals representing 20 tissues will be used in the study.

The resulting data will generate a map that complements other large-scale efforts that are linking our knowledge of gene sequence and cellular phenotypes: studies involving DNA sequencing, SNPs, histone modifications, and transcriptome and proteomic analyses. The epigenome map will be invaluable for understanding gene regulation and the interactions between genes in normal and disease states. It will offer new explanations in well-studied areas such as cancer research, and will also provide a basis for novel approaches to research on environmental effects, nutrition, and ageing (Eckhardt et al. 2004). The HEP also promises to provide DNA methylation markers for disease states, and new targets for drug development and diagnostic applications based on DNA methylation research are already emerging (Cairns et al. 2001). Current efforts to target the epigenomic machinery of cells with drugs have global effects (Besterman and McLeod 2000; Lubbert 2000; Munster et al. 2001), and more refined approaches will become possible with accumulating knowledge in the new field of epigenomics.

## Materials and Methods

### 

#### Tissue samples.

Human tissue samples were obtained from the National Disease Research Interchange (Philadelphia, Pennsylvania, United States) and consisted of tissue material from healthy individuals. Tissue samples included seven different tissue types (adipose, brain, breast, lung, liver, prostate, and muscle) from 32 different individuals (Table S1). DNA was extracted using standard protocols (Sambrook et al. 1989).

#### Bisulphite conversion.

Bisulphite treatment of genomic DNA was performed with minor modifications to a method described previously (Olek et al. 1996).

#### Amplicon design and PCR.

Primers were designed to be at least 21 bases (G + C ≥ 30%) and to contain at least two bases complementary to bisulphite-converted sequence to increase the specificity. To ensure that the primers were not biased for either hypomethylated or hypermethylated sequences, controls were performed using bisulphite-converted unmethylated or in vitro methylated target sequences. PCRs were performed in 96-well plates on MJ Research (Waltham, Massachusetts, United States) thermocyclers in a final volume of 25 μl containing 250 μM dNTPs, 1X PCR Buffer (Qiagen, Valencia, California, United States), 10 pmol each of forward and reverse primer, 1 U *Taq* polymerase (Qiagen), and 8 ng of bisulphite-treated genomic DNA. Water-only controls were also included in each 96-well PCR plate. The cycling conditions were 95 °C for 15 min followed by 40 cycles of 95 °C for 60 s, 55 °C for 45 s, and 72 °C for 90 s, and a final extension step of 10 min at 72 °C. The PCR amplicons for some of the genomic regions that displayed heterogeneous levels of CpG methylation were sub-cloned using the pGEM-T Easy Vector System according to the manufacturer's instructions (Promega, Madison, Wisconsin, United States). The clones were sequenced on ABI 3700 capillary sequencers (Applied Biosystems, Foster City, California, United States) using ABI Prism Big Dye terminator V 3.1 sequencing chemistry.

#### Sequencing.

PCR amplicons were purified using MultiScreen PCR plates (Millipore, Billerica, Massachusetts, United States) and sequenced directly in forward and reverse directions with the same primers used in the PCR. Sequencing was performed on ABI 3700 capillary sequencers using ABI Prism Big Dye terminator V 3.1 sequencing chemistry.

#### Analysis and database generation.

Quantitative methylation rates were estimated from sequence traces using the ESME software (Lewin et al. 2004). This involved appropriate quality control, normalisation of signals, correction for incomplete bisulphite conversion, and mapping of positions in the trace file to CpGs in reference sequences as described in Lewin et al. (2004). Amplicons were mapped to the human genome assembly (NCBI34) using BLAST (Altschul et al. 1990) and CrossMatch (http://www.genome.washington.edu/UWGC/analysistools/Swat.cfm). Using these offsets the positions of CpG dinucleotides were determined in genomic coordinates. Positional data were then loaded into an LDAS database (http://www.biodas.org) suitable for serving to applications using the distributed annotation system (DAS) XML format (Dowell et al. 2001). HEP methylation data converted to DAS format could then be incorporated dynamically into any third-party applications (such as ENSEMBL) capable of understanding DAS. A Web-based, ENSEMBL-like genome browser, driven entirely from DAS data, was created for displaying HEP data and is publicly available at http://www.epigenome.org. The data reported in the pilot HEP study are subject to the HEP's data release policy (http://www.sanger.ac.uk/PostGenomics/epigenome/drp.shtml).

Kolmogorov-Smirnov tests confirmed that the methylation data did not follow Gaussian distribution (*p* <0.0001 in all cases). We therefore used the Mann-Whitney *U* test (a non-parametric test that compares the medians of two unpaired groups of samples that do not follow a Gaussian distribution) to perform three different comparative analyses for methylation profiles: (i) upstream versus intragenic amplicons, (ii) within the upstream amplicon category, CpG sites located within the 5′ UTR versus CpG sites not located within the 5′ UTR but still within 2 kb of the first start codon, and (iii) within the intragenic amplicon category, intronic versus exonic CpG sites.

We designed a software package, METHANE, to identify and generate graphical views of MVPs (see Figures 5 and 6). This tool uses the same DAS data source as the Web browser to generate graphical views but adds additional analysis facilities to compare and display relative methylation level differences in pairwise comparisons. METHANE provides options to compare CpG methylation level differences calculated from either simple averages or medians per tissue or per site. METHANE can export data as tabular text output or as images in SVG, PDF, or postscript formats and is available on request from the authors.

#### Epigenotyping by mass spectrometry.

Assays, based on the GOOD assay for DNA methylation analysis (Tost et al. 2003), were established for amplicons displaying differential methylation between tissue types or inter-individual variability in the sequencing effort. In most cases, three MVPs within an amplicon were simultaneously queried and analysed. Assay volumes started at 3 μl for PCR, with 2-μl additions for shrimp alkaline phosphatase treatment, primer extension, and 5′-phosphodiesterase digest, and the addition of 10 μl of alkylation mix in the respective reaction steps. After dilution with acetonitrile, 0.5-μl samples were transferred to a matrix coated MALDI target plate. All liquid handling was carried out with automated liquid-handling robotics (BasePlate, The Automation Partnership, Royston, United Kingdom). All MALDI-MS analyses were performed with positive ion mode detection on Bruker Autoflex MALDI mass spectrometers (Bruker Daltonics, Billerica, California, United States), equipped with target-plate-changing robots.

To ensure accurate quantification and to compensate for the common preferential amplification in bisulphite-treated DNA, triplicate calibration standards from 0% to 100% methylation in 25% increments were included into the analysis. Statistical parameters were defined to compensate for factors complicating quantification by MALDI-MS, such as bad reproducibility of the crystallisation, different rates of ionisation of analytes, and signal-to-signal interactions. Quantitative results were obtained with high accuracy when 200 laser shots were accumulated on a sample spot, and eight preparations accounted for differences in the sample preparations. Thus, in total, a quantitative data point is obtained from the average of 1,600 individual spectra and calibrated with the help of mixtures with a known degree of methylation. Success rates are above 97%, and standard deviation for data points is less than 2%.

#### Comparison of DNA methylation with mRNA expression levels.

The methylation data were compared with data in the Gene Expression Atlas database (Su et al. 2002) that contains human transcript data based on the U95 build of Unigene, Affymetrix U95A chip. We calculated methylation values for each amplicon using the median of the methylation values for each CpG site within that amplicon. A quantile plot indicated a strongly non-normal distribution of the data, specifically, platykurtosis (one of two different kinds of kurtosis) demonstrating a bimodal distribution. Therefore, for the purposes of simplified statistical analysis, amplicons were classified as either hypermethylated or hypomethylated dependent on whether their median methylation value was either greater than or less than 50%, respectively. We performed methylation versus expression comparisons after removing the data that corresponded to multiple probes that gave contradictory expression patterns for the same gene on the U95 microarray. Equality of mean expression levels between hypermethylated and hypomethylated datasets was tested using a Welch two-sample (unpaired) *t*-test.

## Supporting Information

Table S1 Tissues Used in the HEP Pilot Study (63 KB DOC).

Table S2 Upstream Amplicons Included in the Comparative Analysis of DNA Methylation with mRNA Expression (32 KB DOC).

### Accession Numbers

The LocusLink (http://www.ncbi.nlm.nih.gov/projects/LocusLink/) accession numbers for the genes and gene products discussed in this paper are *C2* (LocusID 717), *CYP21A2* (LocusID 1589), *DOM3Z* (LocusID 1797), *HLA-G* (LocusID 3135), *IGF2* (LocusID 3481), *TNXB* (LocusID 7148), and *tumour necrosis factor* (LocusID 7124).

## Abbreviations

CpG: cytosine–guanine dinucleotide
DAS: distributed annotation system
HEP: Human Epigenome Project
*IGF2*: *insulin-like growth factor 2*
MHC: major histocompatibility complex
MALDI-MS: matrix-assisted laser desorption/ionisation mass spectrometry
METHANE: METHylation ANalysis Engine
MVP: methylation variable position
ROI: region of interest
SNP: single nucleotide polymorphism
*TNXB*: *tenascin-XB*
