# Supplementary material for: DNA Methylation Profiling of the Human Major Histocompatibility Complex: A Pilot Study for the Human Epigenome Project
Source: PLoS Biol. 2004 Nov 23;2(12):e405. doi: 10.1371/journal.pbio.0020405 (PMC529316; doi:10.1371/journal.pbio.0020405)
Supplement: Table S1 — (63 KB DOC). [file pbio.0020405.st001.doc]

**Supplementary Table 1: Tissues used in the HEP-pilot study**

| **Tissue** | **Age** | **Origin** |
| --- | --- | --- |
| Breast | 16 | Normal breast reduction |
| Brain | 17 | Normal Frontal from head injury |
| Brain | 21 | Normal, temporal lobe |
| Brain | 25 | Normal, pons |
| Brain | 36 | normal occipital lobe |
| Brain | 54 | Normal Frontal from head injury |
| Brain | 76 | normal frontal cortex |
| Breast | 15 | Normal breast reduction |
| Breast | 16 | Normal breast reduction |
| Breast | 21 | Normal reduction mammoplasty |
| Breast | 32 | Normal, Breast reduction |
| Breast | 33 | Normal, breast reduction |
| Liver | 21 | Normal, MVA |
| Liver | 27 | normal |
| Lung | 70 | normal |
| Lung | 73 | normal (from smoker with previous lung ulcer) |
| Lung | 74 | normal (from smoker with previous lung cancer) |
| Lung | 76 | normal |
| Lung | 59 | normal |
| Muscle | 70 | normal (amputation) |
| Muscle | 79 | normal (amputation) |
| Muscle | 79 | normal (amputation) |
| Muscle | 81 | normal (amputation) |
| Muscle | 88 | normal (amputation) |
| Prostate | 64 | Benign Prostatic Hypertrophy |
| Prostate | 68 | Benign Prostatic Hypertrophy |
| Prostate | 68 | Benign Prostatic Hypertrophy |
| Prostate | 68 | Benign Prostatic Hypertrophy |
| Prostate | 70 | Benign Prostatic Hypertrophy |
| Prostate | 79 | Benign Prostatic Hypertrophy |
| Prostate | 81 | Benign Prostatic Hypertrophy |
| Prostate | 86 | Benign Prostatic Hypertrophy |
