# Supplementary material for: DNA Methylation Profiling of the Human Major Histocompatibility Complex: A Pilot Study for the Human Epigenome Project
Source: PLoS Biol. 2004 Nov 23;2(12):e405. doi: 10.1371/journal.pbio.0020405 (PMC529316; doi:10.1371/journal.pbio.0020405)
Supplement: Supplementary file 2 [file pbio.0020405.st002.doc]

Supplementary Table 2

Liver			Lung			Prostate		
Gene	*ADV	**median methylation	Gene	ADV	**median methylation	Gene	ADV	**median methylation
HLA-F	2146	0	HLA-F	4282	0	HLA-F	1395	0
HLA-A	5791	0	HLA-A	5691	0	HLA-A	3775	0
PPP1R11	454	0	PPP1R11	550	0	PPP1R11	367	0
TUBB	1009	0	TUBB	282	0	TUBB	560	0
LTA	25	0	GTF2H4	84	0	GTF2H4	36	0
NCR3	20	0	NCR3	20	0	NCR3	32	0
HSPA1B	1655	0	HSPA1B	301	0	HSPA1B	525	0
NOTCH4	152	0	NOTCH4	132	0	AGER	64	0
HLA-DQB1	20	0	TAP2	153	0	NOTCH4	41	0
HLA-DMA	329	0	HLA-DMA	1615	0	HLA-DQB1	20	0
HKE2	64	0	ZNF297	160	0	HKE2	108	0
ZNF297	127	0	DAXX	327	0	ZNF297	168	0
DAXX	295	0	HLA-DQB1	20	2	DAXX	173	0
TAP2	20	4	HKE2	141	2	RING1	370	3
GTF2H4	253	5	RING1	499	3	TAP2	20	4
TNF	575	5	RAB2L	172	8	HLA-DMA	265	4
RAB2L	65	8	TNF	178	68	RAB2L	130	4
RING1	1352	22	BAT8	170	85	TNF	113	61
BAT8	57	77	LTA	95	86	LTA	85	73
HLA-DRA	28	83	AGER	3952	87	DDX16	95	86
DDX16	95	90	DDX16	157	90	HLA-DRA	219	91
MSH5	20	94	HLA-DRA	118	97	CDSN	25	92
AGER	171	95	MSH5	20	98	BAT8	91	95
CDSN	20	96	CDSN	20	100	MSH5	20	96

Legend	
red	ADV < 200
green	ADV > 200
yellow	median methylation < 50
blue	median methylation > 50

*ADV: Average Difference value is a measure of the expression level. An ADV < 200 corresponds to less than 3-5 mRNA copies/cell

**median methylation: The median methylation value of the upstream amplicon associated with the gene
